# Shape Evolution of Hierarchical W_18_O_49_ Nanostructures: A Systematic Investigation of the Growth Mechanism, Properties and Morphology-Dependent Photocatalytic Activities

**DOI:** 10.3390/nano6120240

**Published:** 2016-12-14

**Authors:** Guojuan Hai, Jianfeng Huang, Liyun Cao, Yanni Jie, Jiayin Li, Xing Wang

**Affiliations:** School of Materials Science & Engineering, Shaanxi University of Science and Technology, Xi’an 710021, China; GuoJuanH@163.com (G.H.); Yanni_jie@126.com (Y.J.); lijiayin@sust.edu.cn (J.L.); StellarWangwx@126.com (X.W.)

**Keywords:** W_18_O_49_, microstructure, phase transformation, photocatalytic activity

## Abstract

Hierarchical tungsten oxide assemblies such as spindle-like structures, flowers with sharp petals, nanowires and regular hexagonal structures are successfully synthesized via a solvothermal reduction method by simply adjusting the reaction conditions. On the basis of the experimental results, it is determined that the reaction time significantly influences the phase transition, microstructure and photocatalytic activity of the prepared samples. The possible mechanisms for the morphology evolution process have been systematically proposed. Moreover, the as-prepared products exhibit significant morphology-dependent photocatalytic activity. The flower-like W_18_O_49_ prepared at 6 h possesses a large specific surface area (150.1 m^2^∙g^−1^), improved separation efficiency of electron-hole pairs and decreased electron-transfer resistance according to the photoelectrochemical measurements. As a result, the flower-like W_18_O_49_ prepared at 6 h exhibits the highest photocatalytic activity for the degradation of Methyl orange aqueous solution. The radical trap experiments showed that the degradation of MO was driven mainly by the participation of h^+^ and •O_2_^−^ radicals.

## 1. Introduction

Semiconductor photocatalysis has been regarded as an effective resolution to serious environmental pollution and the global energy shortage [1]. So far, numerous studies related to the photocatalytic performance of oxide semiconductor have been carried out. For example, semiconductors such as TiO_2_ [2], ZnO [3], CuO [4], MoO_3_ [5], WO_*x*_ [6], Bi_2_WO_6_ [7] and g-C_3_N_4_ [8] have been used as efficient photocatalysts. Among them, W_18_O_49_, which is a wide band-gap n-type semiconductor, has an unusual defect structure and intense near-infrared photoabsorption [9,10]. As reported before, the morphology plays an important role in determining the properties of materials. Therefore, several methods have focused on the preparation of W_18_O_49_ with various morphologies such as nanowires, nanorods, nanobundles and hierarchical architectures [11,12,13]. However, a 1D structure has the largest proportion among the synthesized W_18_O_49_ structures, because the fastest growth rate of W_18_O_49_ is achieved by linking the WO_6_ octahedra to form linear rows along the *b* axis [14]. 

Compared with 1D and 2D nanostructures, 3D hierarchical architectures hold many advantages, such as a high surface-to-volume ratio, unique pathways for transportation of charge carriers and abundant transport paths for small organic molecules. Therefore, it is necessary to develop an effective and simple route to control the morphology of W_18_O_49_. Furthermore, there are few reports on how the 3D structure of W_18_O_49_ can affect the optical absorption, photoelectrochemical and photocatalytic properties.

In the present work, a facile, additive-free synthesis of W_18_O_49_ with controlled morphology evolution and phase transition under solvothermal conditions is reported. Methyl orange (MO) is chosen as the target pollutant to evaluate the photocatalytic activity of the samples under UV and visible light irradiation. In addition, photoelectric performances of the samples prepared under different solvothermal time are investigated. The possible reasons for enhancing photoelectrochemical activities are clearly explored.

## 2. Experimental Section

### 2.1. Synthesis of Samples

W_18_O_49_ and WO_3_∙0.33H_2_O/W_18_O_49_ were synthesized by solvothermal method with tungsten hexachloride as precursor and isopropanol as solvent. First, 0.05 M WCl_6_ was dissolved into 40 mL isopropanol with magnetic stirring to form the precursor solution. This solution was transferred to a 100 mL Teflon-lined stainless steel autoclave after stirring for 1 h. The autoclave was sealed and maintained at 160 °C for 1 h, 3 h, 6 h, 12 h in a digital oven, respectively. After being naturally cooled down to room temperature, the products were centrifugally separated and washed with ethanol several times to remove impurities, and then dried in vacuum at 60 °C for 3 h.

### 2.2. Material Characterization

The phase composition of the samples were characterized by a powder X-ray powder diffractometer (XRD, Rigaku D/max-2200 PC, Tokyo, Japan) with Cu Ka radiation (λ = 1.5406 Å). The morphology and structure were determined by scanning electron microscopy (FE-SEM, S-4800, Hitachi, Tokyo, Japan) and a transmission electron microscope (TEM, TecnaiG2F20S-TWIN, Hillsboro, OR, USA). The chemical states and ion ratios of the surface were investigated by X-ray photoelectron spectroscopy (XPS, Thermo ESCALAB 250, Waltham, MA, USA). Brunauer-Emmet-Teller surface areas were characterized with the nitrogen adsorption method on the Micromeritics 3020 instrument, Norcross, GA, USA. The UV-Vis diffuse reflectance spectrum (LAMBDA950, PerkinElmer, Waltham, MA, USA) was used in the wavelength range of 200–800 nm to study the absorption range.

### 2.3. Photocatalytic Evaluation

Methyl orange was adopted as a typical pollutant to evaluate the photocatalytic activity of the as-prepared products. 50 mg of the photocatalyst was added to 50 mL MO solution (10 mg∙L^−1^) and dispersed under ultrasonic vibration for 10 min. Before light irradiation, the suspension was magnetically stirred in darkness for 90 min to achieve adsorption equilibrium. A 300 W Hg lamp and 500 W Xe lamp were used as the light source, which were conducted in an BL-GHX-V photochemical reactor (Bilon Machine Factory, Xi’an, China). The concentration of MO was determined on a UV-Vis spectrophotometer (Unico, UV-2600, Shanghai, China) by monitoring its characteristic absorption at 463 nm.

### 2.4. Photoelectrochemical Measurements

Photoelectrochemical measurements were carried out using a three-electrode quartz cell on the CHI-660B electrochemical system (Xi’an, China). The platinum wire is the counter electrode and the saturated calomel electrode (SCE) is the reference electrode. The tungsten oxide film electrodes were suspended in the 0.5 mol∙L^−1^ Na_2_SO_4_ aqueous electrolyte as working electrodes. The typical working electrode was prepared as follows: 2 mg sample was mixed with 0.5 mL nafion and 1 mL ethanol solution to make slurry. The slurry was then dispersed onto a 4 cm × 2 cm FTO glass and then the FTO glass was dried at 60 °C for 10 h to obtain the electrode. All the photoelectrochemical measurements were performed under a UV light source (CEL-HXUV300, 200–400 nm, Beijing, China). Electrochemical impedance spectra (EIS) were obtained in the frequency range of 0.01–100,000 Hz and then interpreted using a nonlinear least-squares fitting procedure using a commercial software (ZsimpWin, CeAulight, Beijing, China). 

## 3. Results and Discussion

**Figure 1:**
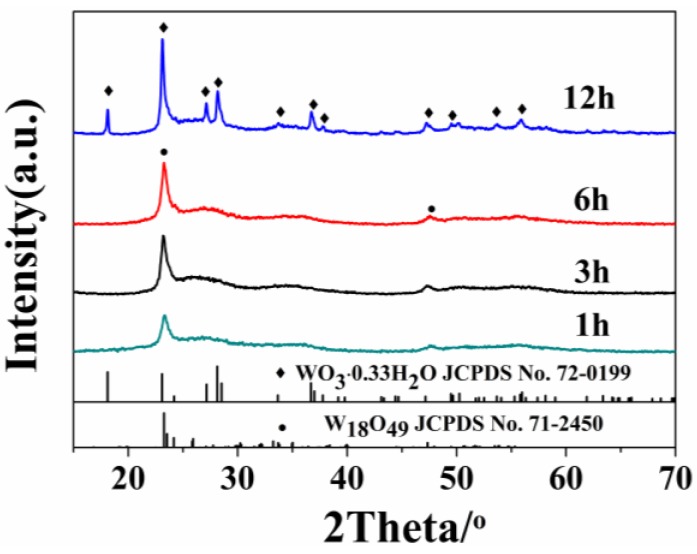
X-ray powder diffractometer (XRD) patterns of the products prepared under different solvothermal reaction times.

The XRD patterns of the products prepared at different reaction time are shown in Figure 1. The sample prepared at first 6 h gives rise to similar XRD pattern that can be indexed to monoclinic W_18_O_49_ phase (JCPDS Card No. 71-2450). The diffraction peaks at 23.48° and 48.02° are indexed to the (010) and (020) planes. This indicates that the crystal growth direction of W_18_O_49_ should be (010), which is consistent with the previous report [15]. When the reaction time reaches 12 h, the XRD peaks of orthorhombic WO_3_∙0.33H_2_O (JCPDS Card No. 72-0199) is found, revealing the existence of a phase transition from monoclinic W_18_O_49_ to orthorhombic WO_3_∙0.33H_2_O. However, whether there is W_18_O_49_ phase or not will be further discussed. The XRD result means that the reaction time exerts a great influence on the crystal phase of the products.

**Figure 2:**
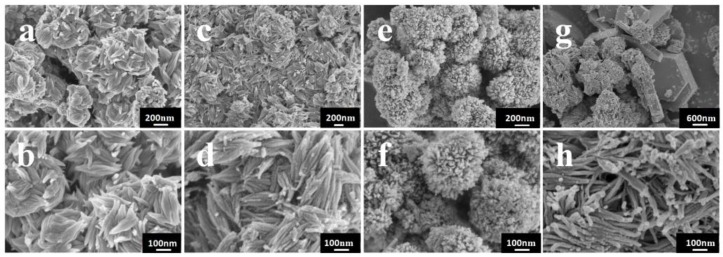
Scanning electron microscopy (SEM) images of the products prepared under different solvothermal reaction times: (**a**,**b**) 1 h; (**c**,**d**) 3 h; (**e**,**f**) 6 h; (**g**,**h**) 12 h.

The morphologies of the samples were investigated by SEM. As shown in Figure 2, the reaction time can largely affect the morphologies of the samples. Figure 2a,c shows the low-magnification SEM images, presenting an overview of the samples prepared at short reaction times (1–3 h). It can be seen that the as-synthesized samples exhibit homogeneous spindle-like particles with width about 50 nm and length in the range of 100–200 nm. Figure 2b,d shows the higher magnification SEM images. It can be seen that all of the spindle-like structure consist of some nanowires which grow along the same direction. Compared with the samples prepared at 3 h, the samples prepared at 1 h are stacked together and there are a small number of irregular particles. According to the results of XRD pattern analysis, the formation of the irregular particles can be attributed to the low crystallinity and some particles could not assemble under the short reaction time. When the reaction time is controlled at 6 h, great changes occur in the morphology of the sample. As shown in Figure 2e,f, the detailed morphology of W_18_O_49_ is a kind of well-defined flower-like structure with diameters in the range of 250–300 nm. The morphology observation indicates each flower-like hierarchical structure is composed of numerous nanowires. The formation of hierarchical structure is attributed to the Ostwald ripening and self-assembly process [16]. As shown in Figure 2g, the regular hexagonal structure is obtained when the reaction time arrives at 12 h, showing the diffusion and rearrangement of the atoms. In Figure 2h, the uniform nanowires also indicate the breaking of the former structures. These two different structures further suggest that the product prepared at 12 h consists of two phase composition.

**Figure 3:**
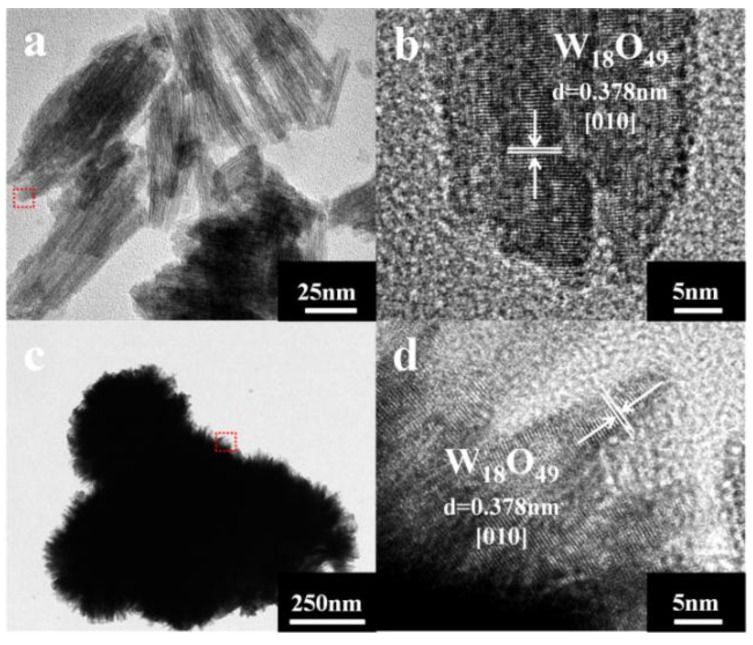
Transmission electron microscope (TEM) images of the products prepared with different reaction times: (**a**) 3 h; (**c**) 6 h. Corresponding selected area electron diffraction (SAED) patterns were obtained at the center of the red rectangles in the TEM images: (**b**) 3 h; (**d**) 6 h.

The crystal structures of samples are further elucidated by TEM analysis. When the reaction time is controlled at 3 h, the spindle-like and nanowire structure can be found in Figure 3a. Figure 3c is a typical TEM image of a flower-like structure with reaction time of 6 h, showing similar morphology as observed in the SEM image (Figure 2e,f). The HRTEM image in Figure 3b,d shows clear lattice fringes. The distances of these fringes are 0.378 nm, in accord with the (010) plane of the monoclinic W_18_O_49_. The TEM analytical results are in agreement with the XRD characteristics. 

**Figure 4:**
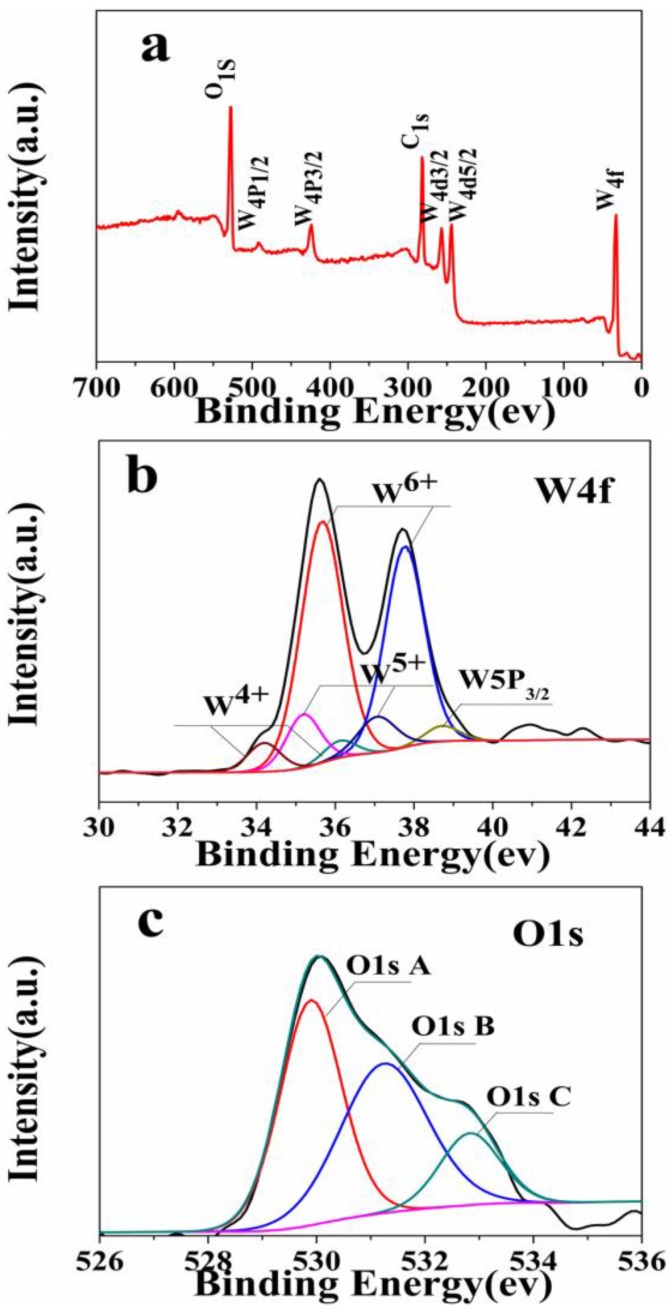
X-ray photoelectron spectroscopy (XPS) spectra of the products prepared at 12 h.

The surface chemical compositions of the sample prepared at 12 h are analyzed by XPS in Figure 4. The binding energies obtained in the XPS analysis are standardized for specimen charging using C1s as the reference at 284.6 eV [17]. The fully scanned spectrum (Figure 4a) confirms the presence of W, O and C elements in the sample. As shown in Figure 4b, the high-resolution spectrum of W4f can be divided into three pairs. The W4f7/2 and W4f5/2 peaks with larger areas, located at 35.69 eV and 37.77 eV, are attributed to the W^6+^. The binding energies of the W 4f peaks at 35.21 eV and 37.16 eV can be assigned to 4f7/2 and 4f5/2 of W^5+^, respectively. Moreover, two additional small peaks centered at 34.20 eV (W4f7/2) and 36.19 eV (W4f5/2) are attributed to the W^4+^ oxidation state [6,18,19,20]. The presence of lower oxidation state (W^4+^) further confirmed the formation of non-stoichiometry W_18_O_49_ [21]. 

As shown in Figure 4c, the high-resolution O1s spectrum presents three peaks at 529.89 eV, 531.24 eV and 532.83 eV. The peak observed at 529.89 eV corresponds to the lattice oxygen O_2_^−^ bonded to W [22]. The medium binding energy component, centered at 531.24 eV, is described as O^−^ or reduced W^*x*+^-state connected O_2_^−^ species [23]. Moreover, the peak observed at 532.83 eV is related to the OH^−^ group, which comes from the adsorbed H_2_O. Saleem [23] found that the broadening of the O1s spectra and the appearance of a shoulder at higher binding energy values evidently indicated the presence of multiple components. Combined with the analyses of XRD, SEM and XPS, the WO_3_∙0.33H_2_O/W_18_O_49_ can be obtained when the reaction time arrives at 12 h. Considering all the above measurements and corresponding results, the reaction time has an important influence on the phase transition and the microstructure of the samples.

Furthermore, the average crystallite size of the prepared samples can be calculated using the following well-known Scherrer formula [24,25]:

$$D=\frac{k\lambda }{\beta \text{cos}\theta }(k=0.9,\;\lambda =0.154)$$

where *D* is the crystal size of the catalyst, *k* is shape factor, λ is the wavelength of the X-rays and β is the full-width at half maximum of the X-ray peak at the Bragg angle θ. The calculated values of *D* are found to be 11.3 nm, 12.0 nm, 13.7 nm and 19.1 nm for the reaction times of 1, 3, 6 and 12 h, respectively. The crystallite sizes for samples increase significantly as the reaction time is extended. When the reaction time is prolonged, the atoms in solid materials acquire sufficient energy to break bonds with its neighbor atoms and then create some lattice distortion during the motion. Thus, the crystallite size increases in the present studies [26].

**Figure 5:**
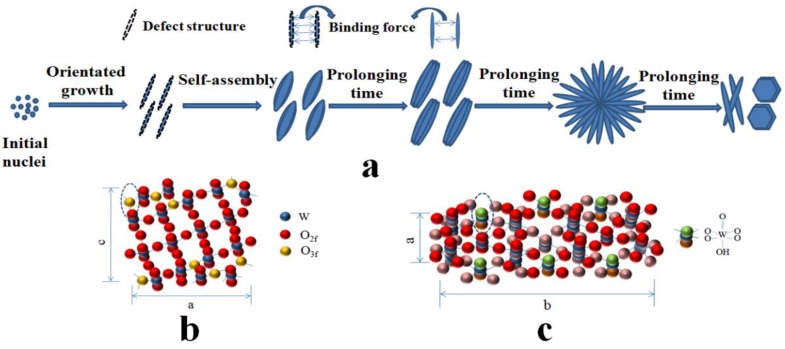
Schematic illustrations: (**a**) the possible formation mechanism of the products obtained at different reaction time; (**b**) the crystal structure of W_18_O_49_; (**c**) the crystal structure of WO_3_·0.33H_2_O.

The crystallinity of samples prepared at 1 h, 3 h, 6 h and 12 h are calculated to be 15.1%, 20.7%, 21.9% and 58.1%, respectively, based on the XRD data. The poorly crystallinity means more defect structures. Moreover, the TEM images in Figure 3 clearly demonstrate that the crystal growth direction of W_18_O_49_ nanomaterials are [010]. According to these experimental results, the possible growth process of the obtained structures can be proposed. At the early stage, WCl_6−*x*_(OC_3_H_7_)_*x*_ firstly formed after WCl_6_ was dissolved in isopropanol solution. Then, the W_18_O_49_ crystal nucleus emerged and formed linear structure. It has been reported that the oxygen vacancies are considered to be very common in tungsten oxides [27,28]. In addition, it has been reported that the oxygen vacancies are generated around a 3-fold coordinative O atom (O3f) in the equatorial O of the octahedral located on the edge of the unit cell and exposed along the b axis of, as shown in Scheme 5b [29,30]. According to the analysis of the TEM, the crystal growth direction of prepared W_18_O_49_ are just along the b axis. Thus, the surface free energy is relatively high. The low crystallinity and exposed surface oxygen vacancies cause high surface free energy for the nanowires structure prepared at short reaction time. To lessen the free energy, these nanowires have strong tendency to coalesce with each other by the lattice free energy and surface free energy, resulting in the formation of spindle-like structure, which is similar with the previous report [31]. At the second stage, the average crystallinity of samples increased gradually with the reaction time increased. It leads to the decreased surface free energy and weaken bonding force between the nanowires. As a result, the nanowires on the spindle-like structure can be clearly seen. When the reaction time is prolonged to 6 h, the surface free energy continues decrease as the improvement of the crystallinity. When the reaction time increases to 12 h, the tungsten oxide hydrates were formed, which indicates that water molecule is present in the reaction system. Consequently, we infer that the increased water molecules inhibit the combination between nanowires. At this stage, the tightly structure scattered into separate nanowires and form the flower-like structure due to the Ostwald ripening and self assembly process [32]. After reaction for 12 h, the nanowires totally dispersed. Meanwhile, the diffusion and rearrangement of the local atomic occurred and part of O–W–O become W=O and W–OH_2_ bonds (Figure 5c), which is attributed to the increased water molecules [33,34]. The possible schematic illustration of the formation of the products obtained at different reaction time is depicted in Figure 5a.

**Figure 6:**
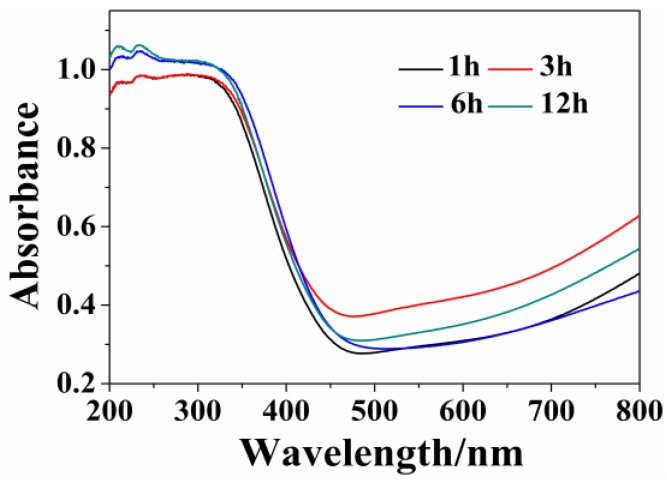
UV-Vis diffuses reflectance spectra of the products prepared under different solvothermal reaction times.

Considering that light absorption plays a key role in determining the photocatalytic activity of semiconductors [35], UV-Vis diffuse reflection spectra are performed. As shown in Figure 6, all of the samples show strong ultraviolet light absorption with an absorption edge around 464–480 nm. The absorption intensity in the region of 200–400 nm of samples gradually increases in the order of W_18_O_49_ (1 h) < W_18_O_49_ (3 h) < W_18_O_49_ (6 h) < WO_3_∙0.33H_2_O/W_18_O_49_ (12 h). In the region of 500–800 nm, the sample prepared at 3 h shows the strongest absorption performance. It is known that the optical absorption of a semiconductor is an important factor determining its photocatalytic performance. The enhanced light absorption may lead to forming more electron-hole pairs. However, whether the light absorption has an important influence on the photocatalytic performance needs further discussion.

**Figure 7:**
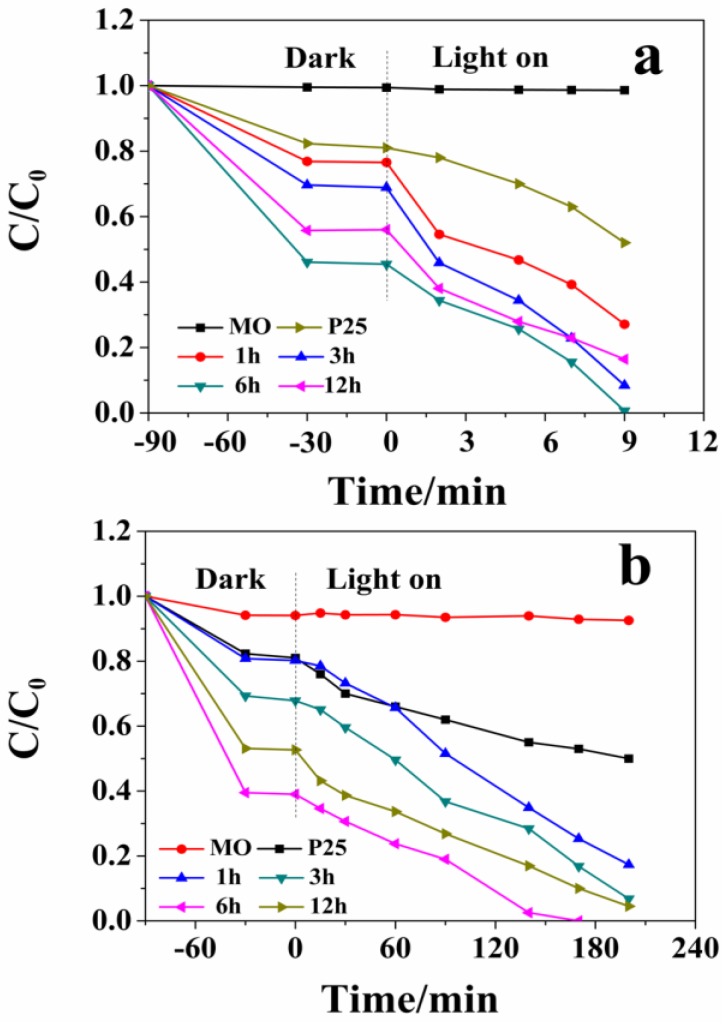
Photocatalytic properties of the products prepared under different solvothermal reaction times: (**a**) UV-light irradiation; (**b**) visible-light irradiation.

As presented in Figure 7, the photocatalytic activity of as-prepared samples are evaluated by measuring the degradation of MO in an aqueous solution under ultraviolet/visible light irradiation. A control experiment without catalyst indicates that MO is not much reduced during the photocatalytic process. The adsorption test shows that the adsorption-desorption equilibrium between catalysts and MO is achieved after the dark stirring for 90 min. From the data in Figure 7a,b, it can be seen that the degradation efficiency for all samples has continuously improved with the increase of the reaction time. Compared with P25, the as-synthesized samples show better photocatalytic activity. The degradation efficiencies with 9 min are 73.06%, 89.23%, 100.00% and 92.60% for W_18_O_49_ (1 h), W_18_O_49_ (3 h), W_18_O_49_ (6 h), and WO_3_∙0.33H_2_O/W_18_O_49_ (12 h) photocatalysts under ultraviolet light irradiation, respectively. Given the high photocatalytic activity of the sampls under ultraviolet light irradiation, the photocatalytic activity for degradation of MO under visible light irradiation has been further researched. As shown in Figure 7b, it is obvious that W_18_O_49_ (6 h) also exhibits superior photocatalytic activity (100% of MO was decomposed after irradiation for 170 min). However, the sample prepared at 1 h still shows poor photocatalytic activity. Although the samples prepared at 12 h and 3 h show good light absorption under ultraviolet and visible light irradiation, respectively, their photocatalytic activities are moderate compared with other samples. Therefore, it can be concluded that the light absorption intensity and the difference in ultraviolet/visible absorption performance are not the major factor of the enhancement for the photocatalytic activity. 

**Figure 8:**
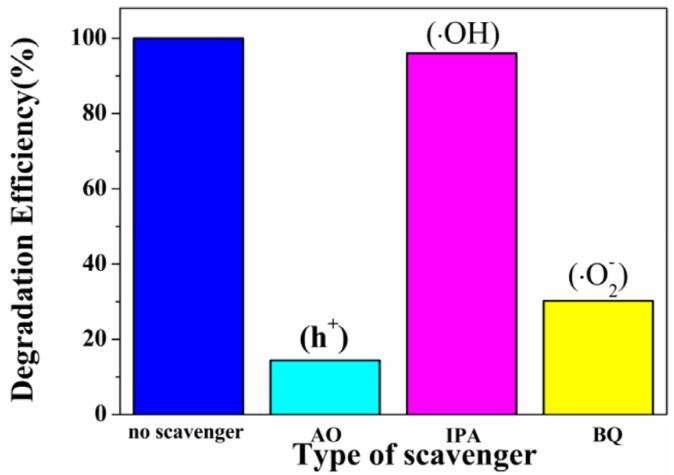
Trapping experiment of active species during the photocatalytic degradation of MO over W_18_O_49_ (6 h) sample under visible light irradiation.

It has been reported that hydroxyl radicals (•OH), superoxide radicals (•O_2_^−^) and active holes (h^+^) are contribute to oxidize organic pollutants. In this study, the 2-propanol (IPA), ammonium oxalate (AO) and benzoquinone (BQ) were added as scavengers for •OH, h^+^ and •O_2_^−^, respectively. The initial concentrations of these scavengers in the reaction system were 1 mmol/L. As can be seen in Figure 8, under visible-light irradiation of the W_18_O_49_ (6 h) photocatalyst, the photodegradation rate of MO had slight decrease after the addition of hydroxyl radical scavenger IPA, indicating that hydroxyl radicals were not main radical species. However, the addition of AO or BQ clearly inhibits the degradation the MO solution. Based on the above results, we can conclude that the degradation of MO was driven mainly by the participation of h^+^ and •O_2_^−^ radicals.

**Figure 9:**
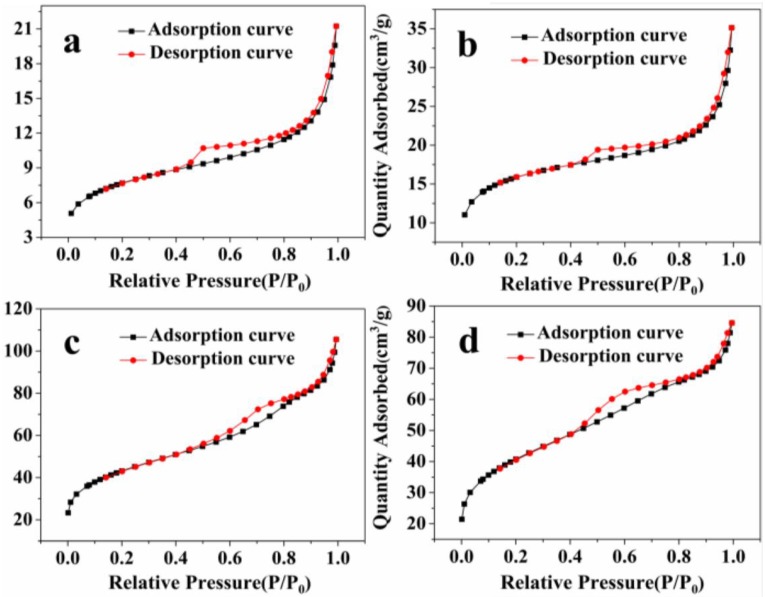
N_2_ absorption-desorption isotherms of the products prepared under different solvothermal reaction times: (**a**) 1 h; (**b**) 3 h; (**c**) 6 h; (**d**) 12 h.

With the extension of reaction time, the dark adsorption performance of samples increase and the W_18_O_49_ prepared at 6 h exhibits superior dark adsorption. In order to explain this, the specific surface areas of as-prepared samples have been investigated using N_2_ adsorption-desorption isotherms, as shown in Figure 9. The BET surface area values of W_18_O_49_ (1 h), W_18_O_49_ (3 h), W_18_O_49_ (6 h) and WO_3_∙0.33H_2_O/W_18_O_49_ (12 h) are 26.5, 53.3, 150.1 and 142.3 m^2^∙g^−1^, respectively. It is generally accepted that the catalytic process is closely related to the adsorption and desorption of molecules on the surface of the catalyst and the dark adsorption is a prerequisite for good photocatalytic activity. A catalyst with a large surface area will provide more active surface sites for adsorption of reactant molecules. Thus, it can be concluded that the enhanced photocatalytic activity of W_18_O_49_ (6 h) is positively correlated with the specific surface area. 

**Figure 10:**
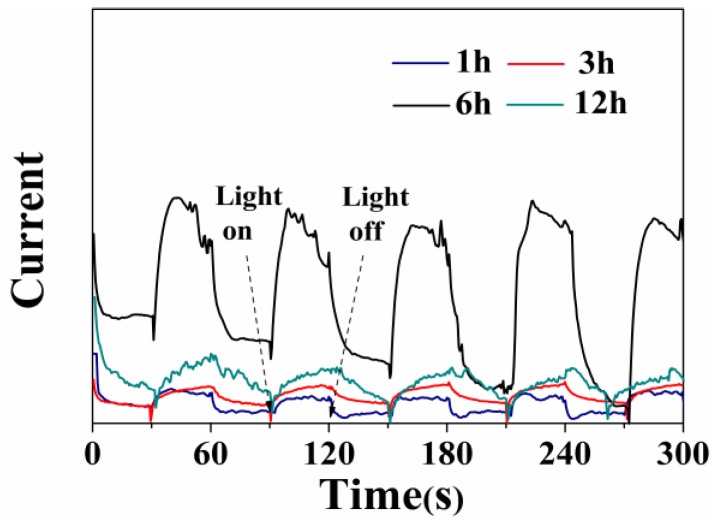
Photocurrent response of the products prepared under different solvothermal reaction times.

To further explain the enhancement of photocatalytic properties, the interfacial charge separation and transfer dynamics of photoelectrons are studied by monitoring the photocurrent-time response, which is correlated with the photocatalytic activity [36]. As shown in Figure 10, the photocurrent responses to illumination are prompt in all cases, but the photocurrent values are different from each other. Moreover, it is worth noting that the separation of photoinduced charge carriers for the sample prepared at 6 h has a notable improvement, demonstrating a positive effect on the separation and transfer efficiency of photogenerated electron-hole pairs [37]. The photocurrent decreases in the order of W_18_O_49_ (6 h) > WO_3_∙0.33H_2_O/W_18_O_49_ (12 h) > W_18_O_49_ (3 h) > W_18_O_49_ (1 h) under light irradiation. 

**Figure 11:**
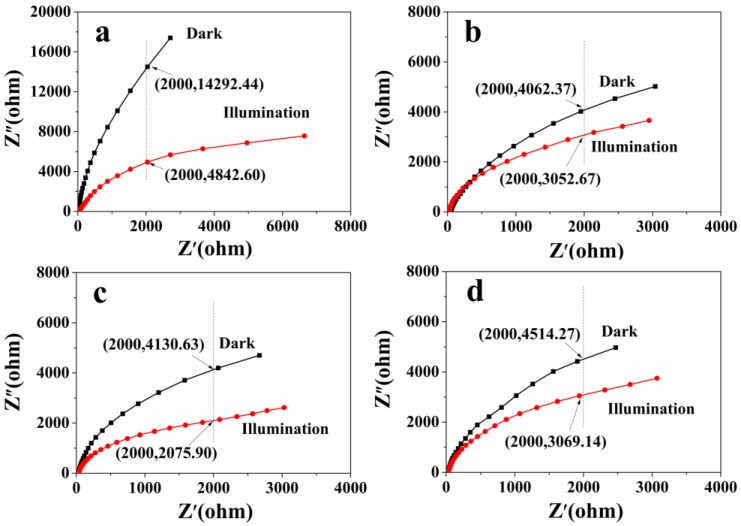
Electrochemical impedance spectra (EIS) Nyquist plots of the products prepared under different solvothermal reaction times: (**a**) 1 h; (**b**) 3 h; (**c**) 6 h; (**d**) 12 h.

The electrochemical impedance measurements of samples with and without light irradiation are shown in Figure 11. It can be observed that the irradiation light causes the small arc radius for all samples, meaning an effective separation of photogenerated electron-hole pairs [38]. The coordinates presented in Figure 11 are used to illustrate the relative value and the change of impedance under ultraviolet light irradiation. According to the coordinates, it is clearly observed that the arc radius of the sample prepared at 1 h has the largest decrease, but it still exhibits the greatest impedance under light irradiation. When lit up, the sample prepared at 6 h shows the smallest impedance arc radius, representing the smallest charge transfer resistance and the highest transfer efficiency of the photogenerated charges in comparison to the other samples. 

The results of the transient photocurrent and electrochemical impedance measurements are consistent with the photocatalytic activities of the as-prepared samples. The data clearly demonstrate that the prepared flower-like hierarchical structure is a good catalyst to remove the MO dye under ultraviolet/visible light irradiation. The good efficiency of the W_18_O_49_ (6 h) catalyst is attributed to the unique hierarchical structures, which can provide radial microchannels for reactant diffusion and thus reduce electron loss [39,40]. Consequently, it exhibits excellent photocatalytic activity. Under the identical conditions, the W_18_O_49_ prepared at 1 h shows poor photocatalytic activity and electrochemical properties, which is mainly related to the low crystallinity. This is because a poorly crystallized crystal with defects always leads to the recombination of photogenerated electrons and holes at defect positions [41]. 

## 4. Conclusions

In summary, W_18_O_49_ with controllable morphologies has been successfully prepared via a facile solvothermal method by simply adjusting the reaction conditions. It is found that the reaction time significantly influences the phase transition, microstructure and photocatalytic activity of the prepared samples. The particle shapes evolve with reaction times from homogeneous a spindle-like structure to a flower-like hierarchical structure, and to a mixture of nanowires and regular hexagonal structure. Furthermore, the driving force of this morphology evolution was found to correspond to the decreased surface free energy and increased water molecules. Moreover, the flower-like W_18_O_49_ catalyst shows excellent photocatalytic activity for MO degradation compared to other samples under UV and visible light irradiation. The radical trap experiments showed that the degradation of MO was driven mainly by the participation of h^+^ and •O_2_^−^ radicals. The enhanced photocatalytic activities of W_18_O_49_ (6 h) can be ascribed to the high BET surface area (150.1 m^2^∙g^−1^) and special hierarchical structures, which possess rich active sites and are beneficial to reduce the recombination rate of the photogenerated electrons and holes.
